# Composite Membranes Based on Functionalized Mesostructured Cellular Foam Particles and Sulfonated Poly(Ether Ether Sulfone) with Potential Application in Fuel Cells

**DOI:** 10.3390/membranes12111075

**Published:** 2022-10-30

**Authors:** Natalia A. Agudelo, Claudia E. Echeverri-Cuartas, Betty L. López

**Affiliations:** 1 Grupo de Investigación e Innovación en Formulaciones Químicas/Escuela de Ingeniería y Ciencias Básicas, Universidad EIA, Calle 23 AA Sur Nro. 5-200, Kilómetro 2+200 Variante al Aeropuerto José María Córdova, Envigado 055428, Antioquia, Colombia; 2 Grupo de Investigación en Ingeniería Biomédica (GIBEC)/Escuela de Ciencias de la Vida, Universidad EIA, Calle 23 AA Sur Nro. 5-200, Kilómetro 2+200 Variante al Aeropuerto José María Córdova, Envigado 055428, Antioquia, Colombia; 3 Grupo de Ciencia de los Materiales/Facultad de Ciencias Exactas y Naturales, Universidad de Antioquia, Dirección: calle 67 No. 53-108, Medellín 050004, Antioquia, Colombia

**Keywords:** proton exchange membranes, sulfonated poly(ether ether sulfone), mesostructured cellular foam, amino groups, sulfonic group, proton conductivity

## Abstract

Composite polymeric membranes were designed based on sulfonated poly(ether ether sulfone) (sPEES) and mesostructured cellular foam (MCF) silica nanoparticles functionalized with organic compounds. Parameters such as molecular weight (MW) of the polymer, nature of the functional group of the MCF silica, and percentage of silica charge were evaluated on the final properties of the membranes. Composite membrane characterization was carried out on their water retention capacity (high MW polymer between 20–46% and for the low MW between 20–60%), ion exchange capacity (IEC) (high MW polymer between 0.02 mmol/g–0.07 mmol/g and low MW between 0.03–0.09 mmol/g) and proton conductivity (high MW polymer molecular between 15–70 mS/cm and low MW between 0.1–150 mS/cm). Finally, the membrane prepared with the low molecular weight polymer and 3% wt. of functionalized silica with sulfonic groups exhibited results similar to Nafion^®^ 117.

## 1. Introduction

Currently, the most common sources of energy generation are mainly related to nuclear and fossil fuels through combustion processes. Nevertheless, the search for new alternatives to the fuel sources of current systems has been one of the main interests in recent years, not only for environmental reasons (generation of nuclear waste, carbon monoxide, and dioxide) [1,2,3] but also due to the decrease in hydrocarbon sources [4,5,6,7].

Among the cleanest energy generation technologies are fuel cells [2], which present high conversion efficiency with low emissions of pollutants into the environment [3,4,8,9,10,11]. Fuel cells are electrochemical devices that directly convert the chemical energy stored in a fuel (such as hydrogen, methanol, and ethanol, among others) into electrical energy. The electron flow in a fuel cell is controlled through electrochemical reactions, and both the fuel and the oxidant (oxygen or air) are kept separated [4,5,11,12,13].

There are different types of fuel cells depending on the electrolyte used. However, proton-exchange membrane fuel cells (PEMFC) are the most used cells [3] since they are one of the cleanest promising technologies [14] and they also present a high energy conversion efficiency [6]. Among the most outstanding applications of these types of cells is transportation, due to its potential impact on the environment (control of greenhouse gas emissions), and stationary and portable power generation [4,11,15].

PEMFC cells employ a proton exchange membrane (PEM) as the electrolyte with high proton conductivity, which defines many of the functions of PEMFC [16,17,18] One of the most important characteristics is that they must have high proton conductivity. Simultaneously, it must act as a barrier and prevent the passage of fuel and oxygen to prevent their direct combustion [6,16]. Finally, they must be electrical insulators to ensure that the electrons generated at the anode flow through the external circuit to the cathode [12,19].

In addition to the characteristics already mentioned, the PEMs must meet other requirements that allow greater efficiency within the fuel cells, among which are: high mechanical [8], thermal, and chemical stability [8], electrochemical stability under operating conditions [8,20], low water permeability [21], humidity control inside the cell [20], dimensional stability during operation time [21] and production costs compatible with the desired application [1,18,20,21].

The most used ion exchange membrane commercially available and recognized as the standard for this type of PEM is Nafion^®^, developed in the 1970s by DuPont de Nemours & Co., Inc., Wilmington, DE, USA [4,19,22,23]. This membrane has a central chain, whose basic structure is perfluorocarbon, similar to Teflon [3,24], which is responsible for the membrane’s chemical, mechanical, and thermal stability. Additionally, it has side chains with sulfonic groups, which allow the transport of ionic charges through the membrane and give it a hydrophilic character [25]. Due to its amphiphilic composition, this membrane presents a separation in nano-phases between the hydrophobic matrix and the hydrophilic ionic domains in the hydrated state (water-soluble groups covalently attached to a non-polar carbon chain insoluble in water) [23].

The chemical structure of Nafion^®^ provides a good combination of performance and durability; it shows excellent mechanical properties and good conductivity (0.10 S/cm) under fully hydrated conditions [8,26]. However, Nafion^®^ has a high production cost [6,8], and in its ionic form, it is difficult to process and cannot be easily dissolved [23,24,27]. Additionally, the Nafion^®^ membrane for direct methanol applications [3] it presents high permeability to this fuel [28], and for hydrogen cells, it has a high electro-osmotic diffusion coefficient, which leads to anode dehydration and cathode flooding. Therefore, this limitation has encouraged the development of alternative membranes that allow high proton conductivity at low relative humidity and high temperatures [25].

Fluorinated materials, such as Nafion^®^, have higher costs, as was mentioned previously, and complex synthesis processes, allowing non-fluorinated material membranes to emerge as an alternative for PEM [28,29]. Some of the materials used for this purpose are poly(aryl ether) such as poly(ether ether ketone) (PEEK) and poly(ether ether sulfone) (PEES) [1], poly(imides) (PIs), poly(ether imides) (PEIs) [30,31], poly(styrene) (PS) and its derivatives, poly(benzimidazole) (PBI) [17,32] and poly(phosphazenes) [2]. Most of these materials have excellent chemical resistance, high thermo-oxidative stability, low permeability to methanol, and long lifetimes, making them suitable as membranes in PEMFC-type fuel cells [23,30].

Various modifications for these materials have been studied to find the best properties for this type of application. However, these polymers are still restricted for application in fuel cells due to low water retention at high temperatures, which decreases proton conductivity [28,29]. For this reason, membranes composed of sulfonated polymers with hygroscopic inorganic oxide particles have been researched [8,17,28] using SiO_2_, TiO_2_ [33], zeolites, zirconium, and montmorillonite, which can retain water at temperatures above 100 °C [34,35,36]. These inorganic fillers have the advantage of decreasing the permeability of methanol [8], which is another requirement sought to be satisfied, since Nafion^®^ presents high permeability of this fuel.

Inorganic SiO_2_ fillers are the most widely used to improve the water retention of PEMs [29,32]; specifically, ordered and homogeneous mesoporous silicas have been studied due to their high surface area and distribution/well-defined mesopore size [36]. Nano-sized inorganic fillers incorporated in PEM, even though they stabilize the membranes, also decrease proton conductivity. However, if the compatibility between the organic and inorganic phases is improved through the functionalization of the silica surface [17,31,32,33,37], the aggregation of silica particles is avoided and thus prevents the decrease in proton conductivity [17,29,37].

Nanostructured silicas can be functionalized with sulfonic groups on the surface [17,37,38] to obtain thermally stable materials that allow capillary condensation in hydrophilic periodic nanochannels, increasing proton conductivity at low relative humidity and high temperatures [35]. 

A report about the preparation of sulfonated SBA-15 mesoporous silica (SPPSU) membranes explains how these particles act as water reservoirs to improve water retention capacity and as supplementary proton conductors, allowing intermolecular transfer of protons between adjacent sulfonic acid groups well-aligned in one-dimensional cylindrical mesoporous channels [36]. Table 1 summarizes the main characteristics of some composite membranes compared with Nafion 117.

Another alternative is to modify the surface of silica fillers with basic groups to overcome the problems of PEMs mentioned above. Within these basic groups are amines [42,43,44] and heterocycles with nitrogen atoms, which present interaction between the acid groups of the polymer through electrostatic forces and hydrogen bonds. This allows control of excessive membrane swelling due to the decrease in the flexibility of the composite material. Acid-base pair formation is expected to increase the proton conductivity of membranes, especially at anhydrous or low humidity conditions [45]. This type of system has been studied using sulfonated poly(ether ether ketone) (SPEEK) with silica particles modified with dopamine (DSiO_2_). Another of the reported systems that have been studied using groups with a basic character is the PEMs prepared from sulfonated polyimides and mesoporous organosilicates functionalized with diaminodiphenyl ether, obtaining systems with an increase in optimal properties to be used in fuel cell applications [46].

Within the mesoporous siliceous materials, there is a mesostructured cellular foam (MCF) type of silica, which is an interconnected pore structure of large pore sizes, large pore volume (1.5 cm^3^/g), and a high surface area (500–1000 m^2^/g) [47,48]. These structural characteristics lead to a 3D porous system interconnected through narrow windows, which can vary based on the material’s synthesis conditions [49,50].

Based on the above, this research aims to develop membranes from a mixture of sPEES with MCF-type silicas functionalized with sulfonic groups and amino groups independently. We evaluated how the membrane functional properties changed, varying the sPEES molecular weight, the silica functionalization type, and the silica percentage incorporated in the membranes. This research explores the inorganic filler modification to improve the compatibility with the polymer and its effect on the membranes’ functional properties in comparison with the pristine sPEES membrane and Nafion^®^.

## 2. Experimental Section

### 2.1. Materials

Bisphenol A (BPA, 99%), 4,4′-difluorosulfone (DFS, 99%), and pyridine were purchased from Aldrich Chemical Company (St. Louis, MO, USA). 1-methyl-2-pyrrolidinone (NMP), toluene, potassium carbonate (K_2_CO_3_, 99%), THF, methanol, sulfuric acid (95–97%, Merck, St. Louis, MO, USA), acetic anhydride (≥98.5%, Merck, St. Louis, MO, USA), 1,2-dichloroethane (1,2-DCE; ≥99.5%, Merck, St. Louis, MO, USA), and dimethyl sulfoxide (DMSO, Merck, St. Louis, MO, USA), sodium chloride (NaCl, Supelco, St. Louis, MO, USA), sodium hydroxide (BioXtra, ≥98%, Sigma-Aldrich, St. Louis, MO, USA), and Mercaptopropyltrimethoxysilane (≥ 95%, Sigma-Aldrich, St. Louis, MO, USA) were analytical reagents and used without further purification. Poly(ethylene glycol)-*block*-poly(propyleneglycol)-*block*-poly-(ethylene glycol) (EO20PO70EO20, P123) was obtained from BASF SE (Ludwigshafen, Germany). Tetraethyl orthosilicate (TEOS, 99%), concentrated hydrochloric acid (37%), toluene, acetone, and ethanol were purchased from Merck. 1,3,5-Trimethylbenzene (TMB, 98+%) and 3-aminopropyltriethoxysilane (APTES, 98%) were purchased from Alfa Aesar (Ward Hill, MA, USA).

### 2.2. Synthesis of Sulfonated Poly(ether ether sulfone) (sPEES)

Polymer synthesis was carried out through a condensation reaction between bisphenol A (BPA) and bisfluorophenyl sulfone (BFFS) (BA:BFFS molar ratio = 1:0.95 and 1:0.99), using a mixture of N-methylpyrrolidone (NMP) and toluene as solvents, and carbonate potassium as a catalyst, as had been reported previously [51]. This reaction was left for 4 h at 150 °C to eliminate the medium’s water through an azeotrope formed between water and toluene. Subsequently, the system was maintained for 16 h at 190 °C. After this reaction time had elapsed, BPA was added in 10% in mol of the initial amount of BPA, and then the reaction was left for 4 h at 160 °C. The polymer solution was filtered and precipitated with a mixture of methanol:water (50:50) and acetic acid. This procedure was carried out to obtain high (HMW-sPEES) and low (LMW-sPEES) molecular weight polymers, varying the stoichiometric imbalance. The solid was washed several times with methanol and allowed to dry at 60 °C.

According to previous reports, PEES polymers were then sulfonated with acetyl sulfate in a molar ratio of PEES:H_2_SO_4_:acetic anhydride = 1:2:2.4, using 106.6 mL of 1,2-dichloroethane as a solvent. This reaction was left at 50 °C for 24 h. The solution obtained was rotoevaporated, then purified by dialysis until reaching neutral pH, and finally, lyophilization was used to obtain the solid material [52].

### 2.3. Synthesis MCF Silica

The MCF silica was synthesized according to the procedure reported previously [53]. An amount of 2.4 g of Pluronic P123 was dissolved in 1.6 M HCl; and then, 1.6 mL of trimethylbenzene (TMB) was added to this final solution. Next, TEOS was added dropwise to the above solution, and the dispersion obtained was left under magnetic stirring for 5 min at 40 °C. This mixture was thermally treated for 20 h at 38 °C without stirring, and then this was transferred to a hydrothermal reactor and left for 24 h at 120 °C. The material obtained was washed with water and left to dry for 24 h at 60 °C. The material was then calcined in an air atmosphere at 600 °C for 5 h at a heating rate of 2 °C/min.

FTIR and TGA were used as additional characterizations to determine the number of silanol groups in each of the materials, as was reported [54]. This characterization was carried out through pyridine adsorption after activating the material at 200 °C for 12 h.

### 2.4. Modifications of Silanol Groups with APTES

The modification of the silica with amino groups was carried out with the precursor APTES (aminopropyl triethoxysilane) using a molar ratio of silanol:APTES = 1:0.5. Briefly, 400 mg of silica was previously activated at 200 °C for 24 h, and subsequently, the silica material was refluxed in 30 mL of toluene with APTES in a nitrogen atmosphere. The reaction was left for 24 h at 110 °C and the product was washed with acetone and ethanol. Finally, the material was dried at 50 °C for two days.

### 2.5. Modifications of Silanol Groups with MPTMS

The modification of the silica with sulfonic groups was conducted using mercaptopropyltrimethoxysilane (MPTMS) as the precursor with a molar ratio of silanol:MPTMS = 1:0.5. The procedure was similar to modify the silica with APTES. An amount of 400 mg of silica was previously activated at 200 °C for 24 h. Then, 30 mL of toluene with MPTMS in a nitrogen atmosphere was added and refluxed for 24 h at 110 °C. The final product was washed with acetone and ethanol, and the solid material was dried using an oven at 50 °C for two days.

### 2.6. Characterization of Polymers and Silica

The Fourier transform infrared (FTIR) spectra (PerkinElmer Spectrum One FTIR; PerkinElmer, Inc., Waltham, MA, USA) of the polymer (before and after sulfonation) and the silica with and without functionalization were recorded between 600 and 4000 cm^−1^ at a resolution of 4 cm^−1^ using 16 cumulative scans. Approximately 2 mg of sample was mixed with KBr and pressed into a thin disk.

The proton nuclear magnetic resonance (^1^H NMR) spectra of the polymers were performed on a Bruker AMX-300 spectrometer (Billerica, MA, USA) operated at 300 MHz. The polymers PEES were dissolved in deuterated chloroform (CDCl_3_) and sulfonated samples were dissolved in DMSO-d_6_, and then the spectra were obtained at 25 °C. Chemical shifts (δ) were expressed in ppm with respect to the CDCl_3_ and DMSO-d_6_ signals, respectively.

Thermal stability of the materials (polymers and silica) was evaluated by using a thermo-gravimetric analyzer (TGA) (TA Instruments model Q500, New Castle, DE, USA) at a heating rate of 10 °C min^−1^ under a nitrogen atmosphere. Differential scanning calorimetry (DSC) measurements were carried out using a temperature-modulated DSC (TM-DSC) TA Instrument Q100. The samples are hermetically sealed in aluminum pans and their thermal history was erased by heating from 25 to 200 °C at 20 °C/min, and then cooling to 20 °C at 20 °C/min. The thermograms of all samples were acquired from 2 to 360 °C heating at 10 °C/min after the thermal history erasing.

To characterize the pore size, total pore volume, and surface area of the MCF silica, according to the Brunauer–Emmett–Teller (BET) theory, nitrogen adsorption–desorption measurements were performed at 77 K using the ASAP 2020 Plus Physisorption System (Micromeritics Instrument Corporation, Norcross, GA, USA). All samples were degassed for 24 h at 100 °C before measurements. The cell size (Dc) and window size (Dw) of the silica MCF were obtained from the peak positions of pore size distribution curves.

Dynamic light scattering (DLS) was used to determine the particle size of the MCF silica at 25 °C by using the LB-550 DLS Nanoparticle Size Analyzer (Horiba, Ltd., Kyoto, Japan). All samples were tested in quadruplet. For these measurements, dispersions of silica (1% wt.) in water were prepared after calcination.

The superficial charges of the silica dispersion before and after functionalization were evaluated according to the ζ-potential at a pH value of 3–11, using a Zetasizer Nano ZS DLS instrument (Malvern Instruments, Ltd., Malvern, England).

### 2.7. Preparation and Characterization of Composite Membranes

The hybrid membranes were made with different percentages of silica with respect to the polymer weight (3%, 6%, and 9%).

Silica was dispersed in 1 mL of DMSO using an ultrasound probe (20 KHz at 90% amplitude for five minutes, 10 s, and 5 s off). Subsequently, the polymer was added, and to achieve silica dispersion in the polymeric matrix, the probe was used again (by 5 min). The dispersion silica–polymer was left in magnetic contact for 12 h. After this time, the dispersions were deposited in a silicone mold and left under a constant flow of nitrogen for 3 days at 70 °C. Subsequently, successive washes with water were performed to remove the residual DMSO from the membranes. These membranes were characterized by TGA and DSC.

After preparing the membranes and making a morphological and structural characterization, the functional characterization (water uptake and ion exchange capacity) of all the membranes and their proton conductivity were compared at different temperatures with the commercial material reference (Nafion^®^ 117).

### 2.8. Water Uptake

The water content in the sulfonated membranes was evaluated for immersing samples in deionized water at 25 °C, 50 °C, and 80 °C and equilibrated for 24 h, 48 h, and 72 h. Then, for each membrane and time, the excess water was removed with filter paper, and the membranes were weighed (*W_h_*) and submerged again in water. Then, the samples were dried at 60 °C (*W_d_*). The water content was calculated from Equation (1) [55,56,57]:

(1) $$WU\;\left(\%\right)=\frac{W_{h}-W_{d}}{W_{d}}\times 100$$

where *W_h_* is the weight (mg) of the hydrated sample at different times and *W_d_* is the weight (mg) of the sample after being dried at 60 °C. The experiment was performed in triplicate.

### 2.9. Ion Exchange Capacity (IEC)

The ion exchange capacity of the membranes was determined by titration. This parameter indicates the number of milliequivalents of ions in 1 g of dry polymer (meq/g). Briefly, each of the sulfonated membranes was equilibrated in a 2.0 M NaCl solution for 72 h at 50 °C. Then, the protons exchanged with sodium ions formed HCl, which was titrated with a standardized 0.01 M NaOH solution using phenolphthalein as an indicator.

The IEC was calculated according to Equation (2), where *V_NaOH_* and *C_NaOH_* are the volume (L) and the molar concentration (M) of the NaOH solution (previously standardized), respectively, and *W_d_* is the weight of the dry sample (g) [57,58].

(2) $$IEC=\frac{V_{NaOH}\times C_{NaOH}}{W_{d}}$$

### 2.10. Protonic Conductivity

The conductivity measurements of the hydrated and activated membranes with a 0.5 M sulfuric acid solution for 72 h were performed in an Autolab PGSTAT302N (Metrohm AG, Ionenstrasse, Switzerland) with a FRA32M module designed for electrochemical impedance spectroscopy. It worked in a frequency range from 1.0 MHz to 0.1 Hz. The sample was placed between two stainless steel discs, and the data were analyzed using the NOVA 1.11 software.

## 3. Results and Discussion

### 3.1. Synthesis and Characterization of Sulfonated Poly(ether ether sulfone) (sPEES)

**Figure 1:**
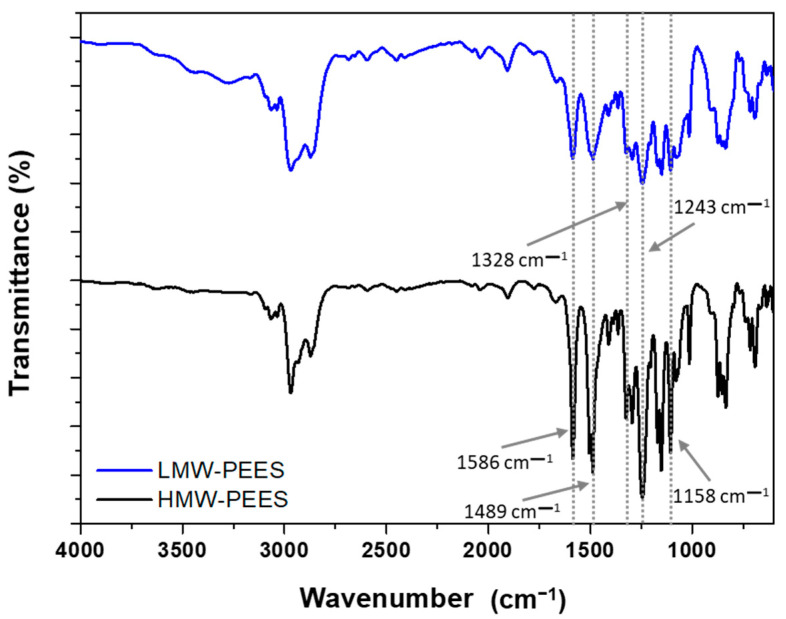
Infrared spectrum of PEES of high (HMW-PEES) and low (LMW-PEES) molecular weight.

**Figure 2:**
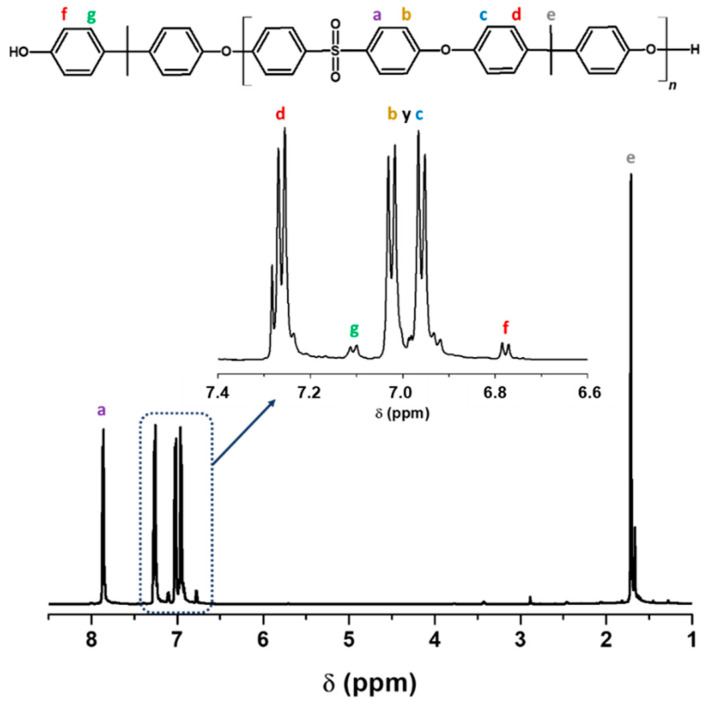
Structure and ^1^H NMR spectrum of LMW-PEES sample.

Two PEES polymers were synthesized using two molar ratios of BA:BFFS = 1:0.95 and 1:0.99 to obtain a low molecular weight polymer (LMW-PEES) and a high molecular weight polymer (HMW-PEES), respectively. To confirm PEES polymer formation, infrared spectroscopy (FTIR) (Figure 1) and proton nuclear magnetic resonance (^1^H NMR) (Figure 2) analyses were performed. In the infrared spectra, characteristic signals of the sulfone group (O=S=O) are observed at 1158 cm^−1^, corresponding to symmetric stretching, and 1328 cm^−1^, corresponding to asymmetric stretching [59]. The signals at 1243 cm^−1^ and 1100 cm^−1^ are associated with asymmetric and symmetric stretching of the C-O-C in the aryl ether group, respectively. The typical stretching signals of the benzene rings occur at 1489 cm^−1^ and 1586 cm^−1^. Additionally, a small signal at 3432 cm^−1^ associated with the presence of terminal hydroxyl groups (phenolics) for the LMW-PEES polymer [60] can be observed.

^1^H NMR carried out the confirmation of the structure of the polymers. Figure 2 shows the spectrum for LMW-PEES where the six protons corresponding to the methyl groups at the ends appear at 1.71 ppm, while the methyl of the polymer structure appears at 1.76 ppm (s, 6H, e). The protons ortho to the sulfone group are found at 7.83 ppm (m, 4H, a). In the case of the protons ortho to the ether bond, they appear between 6.9 and 7.1 ppm (m, 8H, b, c). The ortho protons to the methyl groups are found at 7.25 ppm (m, 4H, d). Finally, the protons of the terminal ring of the structure and which are ortho and meta to the –OH, are between 6.80–6.83 ppm (d, 2H, f) and 7.13–7.16 ppm (d, 2H, g), respectively [61]. The HMW-PEES polymer presented a spectrum like that shown in Figure 2.

The molecular weight of the polymers was determined by ^1^H NMR; through this technique, the aromatic protons located on the terminal phenolic groups can be differentiated from the aromatic protons of the repeating unit. For the determination of *M_n_*, Equation (3) was used [51], comparing the signals of the spectrum:

(3) $$M_{n}=\left(\frac{A_{Ha}}{4}\right)MW_{u.r.\;}+227.3$$

where $A_{Ha}$ is the integration of the peak associated with the aromatic protons in the repeating unit marked as ‘a’ (Figure 2), 4 is the number of aromatic protons ‘a’, $MW_{u.r.\;}$ is the molecular weight of the repeating unit, and 227.3 is the molecular weight of the BPA end group. The integrals used were determined once the integral of the corresponding terminal phenolic protons was adjusted to 1.00. The molecular weight value for the LMW-PEES polymer was 7600 Da, and for HMW-PEES, it was 15,200 Da.

After carrying out the characterization of the polymers, the sulfonation of the polymers in 1,2-DCE as solvent was carried out to reduce the viscosity of the solution. Additionally, the sulfonating agent was prepared with an excess of acetic anhydride to eliminate traces of residual water since this can interfere with the sulfonation process [51]. The introduction of sulfonic acid groups within the structure of homopolymers through post-polymerization methods occurs through electrophilic substitution reactions of aromatic rings.

**Figure 3:**
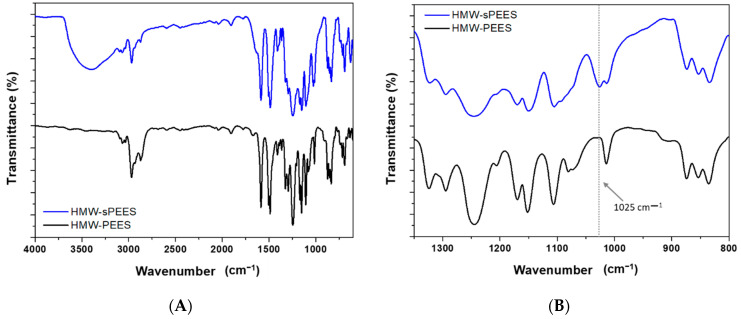
(**A**) Infrared spectrum of the polymer before (HMW-PEES) and after (HMW-sPEES) sulfonation; (**B**) broadening of the infrared spectrum in the region from 1350 cm^−1^ to 800 cm^−1^.

The sulfonation reactions were carried out at 50 °C and 24 h for all the polymers. The sulfonated polymers were characterized by FTIR to confirm the insertion of the sulfonic groups within the aromatic structure of the polymers. In Figure 3, the infrared spectrum for HMW-PEES is presented, and after its sulfonation (HMW-sPEES), the sulfonated LMW-PEES sample (LMW-sPEES) showed the same characteristic bands.

**Figure 4:**
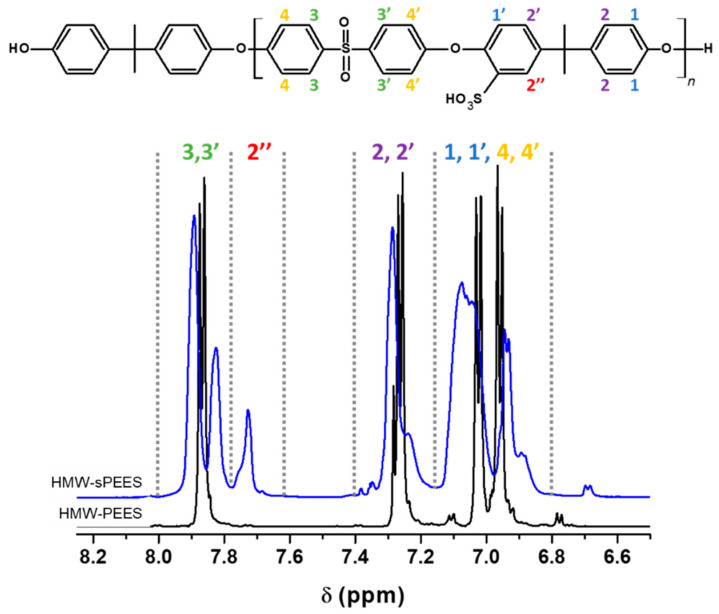
^1^H NMR spectrum of the polymers before (HMW-PEES) and after sulfonation (HMW-sPEES).

For the sulfonated PEES samples, the infrared spectrum, in general, does not show a clear presence of the characteristic bands of the sulfonic acid groups because the sulfone groups (O=S=O) of the main chain of the polymer overlap with the specific signals of the sulfonic acid groups. However, it is observed in Figure 3B that the presence of a shoulder around 1025 cm^−1^ is associated with the extension of -SO_3_ of the sulfonic group [62], which shows the incorporation of these groups in the polymer structure. However, confirmation of the PEES modification was performed by ^1^H NMR (Figure 4). The sulfonated poly(ether ether sulfone) presents a characteristic signal at 7.72 ppm corresponding to the proton adjacent to the sulfonic group (2″ in Figure 4) [63], which confirms the insertion of the sulfonic acid groups in the structure of the polymer.

^1^H NMR spectroscopy allows a quantitative evaluation of the degree of sulfonation as long as there are shifts in the signals of the protons of the polymeric chain due to the presence of the fractions of sulfonic groups. This technique was employed to determine the degree of sulfonation for each sample in DMSO-d_6_. For calculating the degree of sulfonation (*DS*), aromatic signals between 6.5 and 8.0 ppm were used. Equation (4) and Equation (5) were used to determine the *DS* of the PEES, where the signal at 772 ppm was used, which is fully defined and is proportional to the *DS* (mol H^+^/mol repetitive unit) of each of the samples. These equations were previously reported by [64]:

(4) $$R_{1}=\frac{I\left(2^{\prime \prime }\right)}{I\;\left(3,3^{\prime }\right)}\;\;\;;\;\;\;\;\;\;\;\;\;\;\;\;\;\;DS=4\;\times \;R_{1}$$

(5) $$R_{2}=\frac{I\left(2^{\prime \prime }\right)}{I\left(2,\;2^{\prime },\;2^{\prime \prime }\right)}\;\;\;;\;\;\;\;\;\;\;\;\;\;DS=4\;\times \;R_{2}\;\;\;$$

where $I\left(2^{\prime \prime }\right)$ corresponds to the integral of the signal marked as 2″ in Figure 4 and is proportional to the degree of sulfonation of each sample; $I\;\left(3,3^{\prime }\right)$ corresponds to the integral of the protons 3 + 3′; and $I\left(2,\;2^{\prime },\;2^{\prime \prime }\right)$ corresponds to the integral of the proton 2″ plus the integral of the protons 2 + 2′. Table 2 shows the assignment of protons in the polymer structure and the number of protons corresponding to each of these positions. *DS* values for the HMW-sPEES and LMW-sPEES were 75.7% and 76.1%, respectively.

### 3.2. Synthesis of MCF Silica

The synthesis of MCF silica particles was previously reported and the results obtained concerning their porous properties and particle size are presented in Table 3.

**Figure 5:**
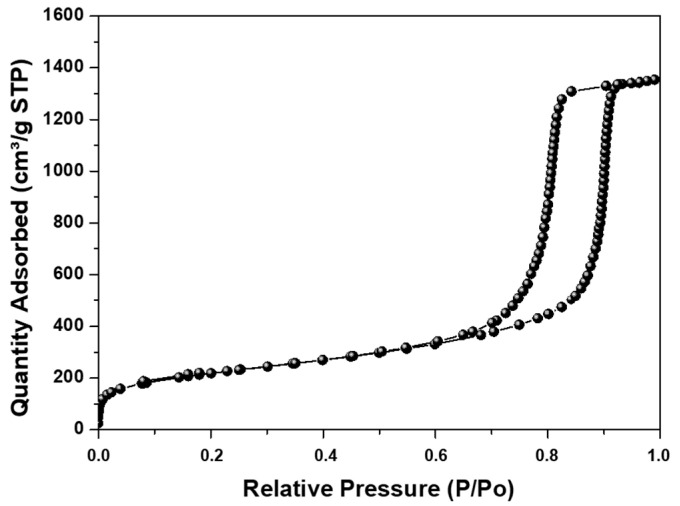
Nitrogen adsorption–desorption isotherm of MCF-type silicas.

The sample presented a high surface area with type IV isotherms (Figure 5), indicating that mesoporous materials with MCF-type morphology were obtained. This is evidenced by obtaining size distributions for the cell (Dc) and the window (Dw), which are large cage-like cellular pores connected by windows, as was previously reported [48,49].

The number of silanol groups was determined following the methodology proposed by [54] and was found to have a value of 2.33 silanol/nm^2^. This value was used for silica surface modification using APTES and MPTMS.

### 3.3. Modifications of Silanol Groups with APTES and MPTMS

After modifying the silica surface with amino groups using APTES and with sulfonic groups using MPTMS, FTIR characterization was carried out to verify the presence of these functional groups in the silica particles.

**Figure 6:**
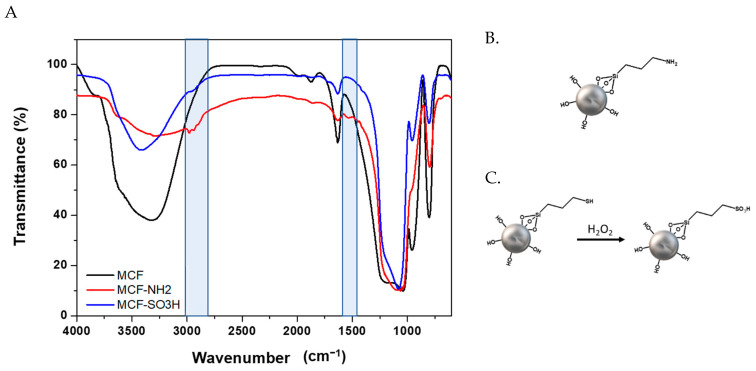
(**A**) Infrared spectrum of MCF silica before and after modification with APTES; (**B**) scheme of silica modified with APTES (MCF-NH_2_); and (**C**) with MPTMS (MCF-SO_3_H).

Figure 6A shows the infrared spectrum before and after modification with APTES (Figure 6B) and MPTMS (Figure 6C). In the region from 2500 cm^−1^ to 3000 cm^−1^ and between 1600 cm^−1^ and 1300 cm^−1^, characteristic signals of C-C and C-H bonds of APTES and the bending and stretching of bonds of the methylene groups of MPTMS are observed [50,65]. These signals are not present in the starting silica, confirming that surface modification with the organic compounds occurred.

Specifically, for the silica modified with MPTMS, in those cases in which complete oxidation of the -SH groups are not achieved, a signal should be observed in the infrared spectrum at 2550 cm^−1^ [66]. However, this signal was not evidenced for the samples modified with sulfonic groups via the oxidation process [67].

**Figure 7:**
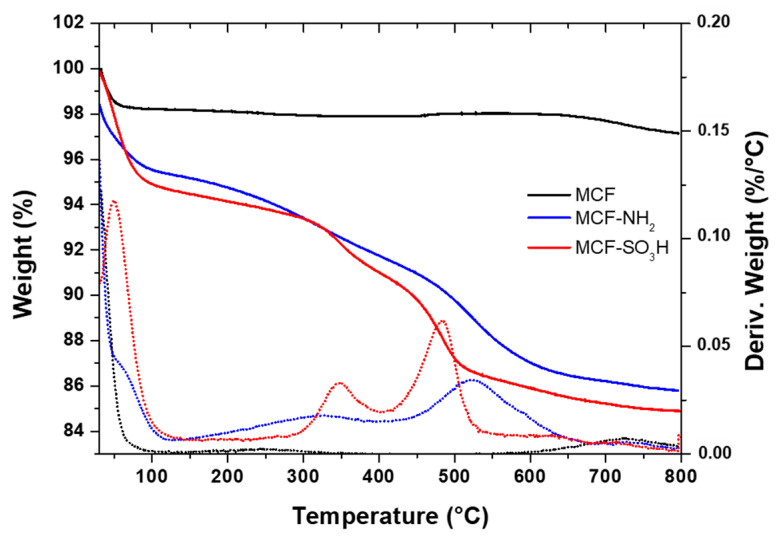
Thermogram of silica MCF before and after modification with APTES (MCF-NH_2_) and MPTMS (MCF-SO_3_H).

The TGA results (Figure 7) show two losses in mass with increasing temperature for both samples. The first loss, below 200 °C, corresponds to the desorption of water molecules (dehydration), which occurs for all materials. In the case of the modified sample with amino groups, it could also be associated with the remaining APTES because its boiling point is 217 °C; therefore, it could be assumed that those physically adsorbed molecules are completely desorbed when reaching 300 °C [68].

The second loss for the sample modified with APTES, between 300 and 600 °C, corresponds to the decomposition of the aminopropyl groups chemically bonded to the silica [66,68]. The temperature at which the maximum decomposition of the material occurs is around 500 °C, where the C-Si bond breaks. For the sample modified with MPTMS, above this temperature, two losses are observed—one between 300 °C and 400 °C and another more pronounced between 400 °C and 520 °C—that correspond to the sulfonic groups incorporated in the structure of silica, which agrees with results reported in the literature for silica functionalized with MPTMS [69]. These two weight losses may be associated with partial oxidation of the mercapto (-SH) groups; thus, the loss between 300 and 400 °C corresponds to the decomposition of the propyl mercapto groups, while the weight loss between 400 and 520 °C corresponds to the thermal degradation of the propylsulfonic acid groups [65].

The amount of APTES and MPTMS grafted to the surface of MCF silica particles was determined considering the weight loss above 300 °C. The amount by weight of APTES was determined to be 9.0%, while for MPTMS it was 9.1%.

**Figure 8:**
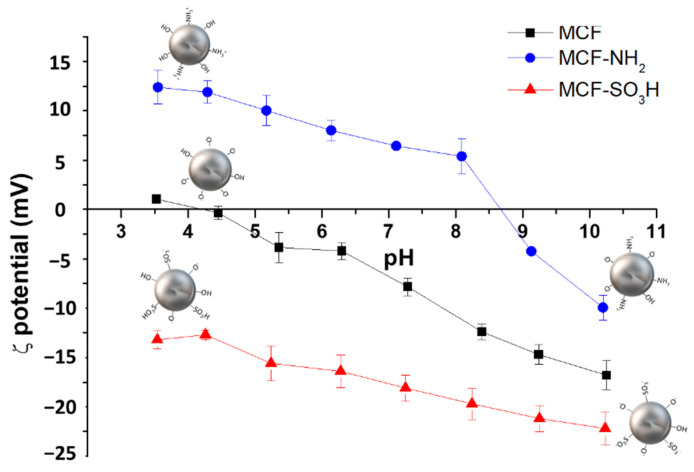
ζ-potential curves as a function of pH for MCF silicas before and after functionalization with APTES (MCF-NH_2_) and MPTMS (MCF-SO_3_H).

In addition to the percentage of loss of organic material in the silica particles that, together with the FTIR analyses, show the modification of these siliceous materials, the evaluation of the surface charge of the particles was carried out through ζ potential measurements in the function of pH, for the modified and unmodified silica. Figure 8 shows that the ζ potential of the materials before and after the modification with APTES and MPTMS strongly depends on pH.

For unmodified silica, the ζ potential had a negative value throughout the pH range. In contrast, the samples modified with amino groups had positive ζ potential values in a pH range of 3.5 to 9. This behavior is due to the unmodified silica having silanol groups on the surface of the particle, which, due to their acid character, tend to deprotonate, forming Si-O^−^ species that are responsible for the negative charge of the unmodified silica throughout the range of pH evaluated. When silica is modified with amino groups, a change occurs in the surface charge of the particles due to the substitution of silanol groups (-Si-OH) by amino groups (Si-NH_2_), which have a basic character; therefore, these materials at acidic pH are protonated, obtaining -NH_3_^+^ species, which explains the positive values of the ζ potential for materials modified with APTES [70,71].

When comparing the results of the unmodified system with those modified with MPTMS, it was observed that the surface charge changes as a function of pH, being more negative (in the entire pH range studied) for those systems that have sulfonic groups in their structure. This negative charge increases as the pH increases due to the deprotonation processes of the sulfonic acid groups, which generates a negative charge on the silica particles. This behavior confirms that the MPTMS was incorporated into the silica structure and that it is in its oxidized form (-SO_3_H).

The modified materials were characterized concerning their porous properties, which are presented in Table 4, where it is observed that the surface area decreases with the incorporation of amino groups in the silica structure, as has been reported by other authors [50].

**Figure 9:**
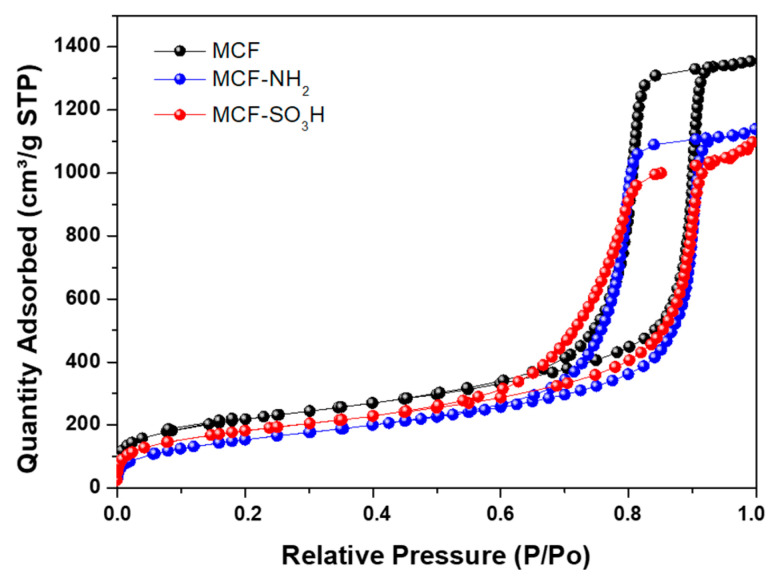
Nitrogen adsorption–desorption isotherms of MCF silica before and after modification with APTES (MCF-NH_2_) and MPTMS (MCF-SO_3_H).

From the isotherms presented in Figure 9, it can be seen that after the modification, the modified materials retain the structure of the MCF silica with pores (cells) connected (through windows), which, additionally, presented absorption isotherms IV, as described for the unmodified MCF.

### 3.4. Preparation and Characterization of Composite Membranes

The sPEES-silica composite membranes were prepared according to the previously described procedure, with both the low molecular weight polymer (LMW-sPEES) and the high molecular weight polymer (HMW-sPEES); both polymers were used to evaluate whether the molecular weight of the polymer affects the physical and chemical properties of the final system. Additionally, to seek an increase in the functional properties of these membranes, modified silica with amino groups and modified silica with sulfonic groups were added in order to compare and determine in which of the systems there is an improvement in the structural and functional properties of the membranes.

**Figure 10:**
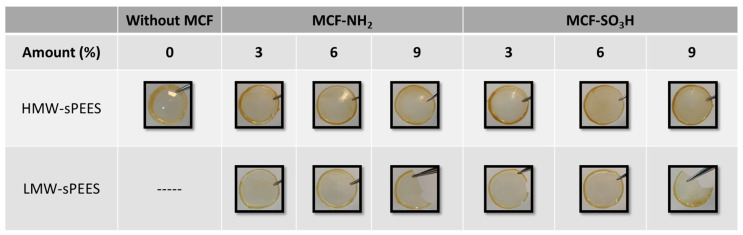
Images of composite membranes prepared from A. HMW-sPEES and B. LMW-sPEES with MCF modified with -NH_2_ and -SO_3_H groups.

Figure 10 shows the images of the membranes obtained for the four systems: HMW-sPEES x% Si-NH_2_, HMW-sPEES y% Si-SO_3_H, LMW-sPEES x% Si-NH_2_, and LMW-sPEES y% Si-SO_3_H, where ‘x’ corresponds to the theoretical percentage of silica MCF-NH_2_ and ‘y’ to the theoretical percentage of silica MCF-SO_3_H incorporated in the membrane.

For the membranes prepared from the LMW-sPEES polymer and a higher percentage of silica, it was observed that these membranes were more fragile than those prepared with HMW-sPEES, which may be related to the differences in molecular weight. It has been reported that polymers with lower molecular weight have lower mechanical properties [72]; this is because a molecular weight is required to promote molecular ‘entanglements’ (critical molecular weight), which allow for the formation of a pseudo network that leads to obtaining a more helpful material for structural applications.

On the other hand, the high percentage of silica can lead to the formation of aggregates that prevent a good dispersion of the particles in the polymer. Therefore, it is possible that aggregates of inorganic particles are formed that can act as stress generators, thus leading to the fracture of the material [73]. Additionally, it is possible that there is a greater restriction in the mobility of the polymer chains by increasing the amount of inorganic material in the membranes [74], favoring the fragility of the system.

**Figure 11:**
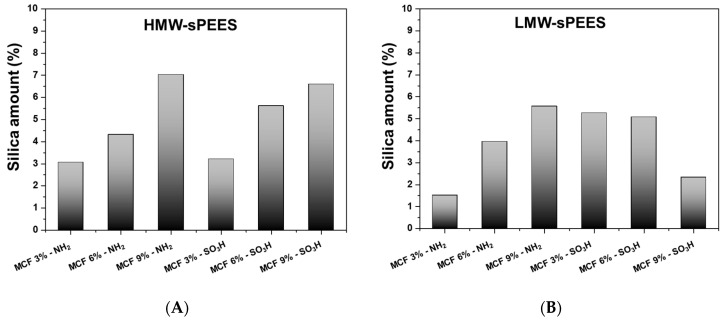
Final silica content MCF-NH_2_ and MCF-SO_3_H in the membranes of (**A**) HMW-sPEES and (**B**) LMW-sPEES.

The amount of inorganic material that remained incorporated in the membranes was quantified through TGA analysis. The results are presented in Figure 11, where it is observed that for most of the systems, as the theoretical content of particles of silica, there is an increase in the real content of this inorganic material in the membranes. However, for the membranes prepared from LMW-sPEES with MCF-SO_3_H, it was observed that the real content of silica in the membranes began to decrease as the theoretical amount increased. This behavior could somehow reflect that there was little dispersion of the inorganic material within the polymeric matrix as the load of modified silica with sulfonic groups increased. This could perhaps be due to the fact that the silica, having a negative charge on the surface, could present electrostatic repulsions with the sulfonic groups of the polymer, leading to the formation of aggregates that were not well dispersed in the polymeric matrix.

The thermal properties of the membranes were evaluated through differential scanning calorimetry (DSC); for all the membranes, a T_g_ value of 189 °C was obtained, which corresponds to the T_g_ value for the sulfonated polymer, indicating that the incorporation of silica particles did not affect the segmental mobility of the polymer after preparing the membranes. Possibly, the amount of silica incorporated in the polymeric matrix was too low to generate changes in the T_g_ value. It has been reported that membranes with silica percentages greater than 20% begin to show an increase in the T_g_ value due to the generation of a rigid region of the polymer due to the tensions that arise during the formation of membranes in the polymer–silica interface [75].

**Figure 12:**
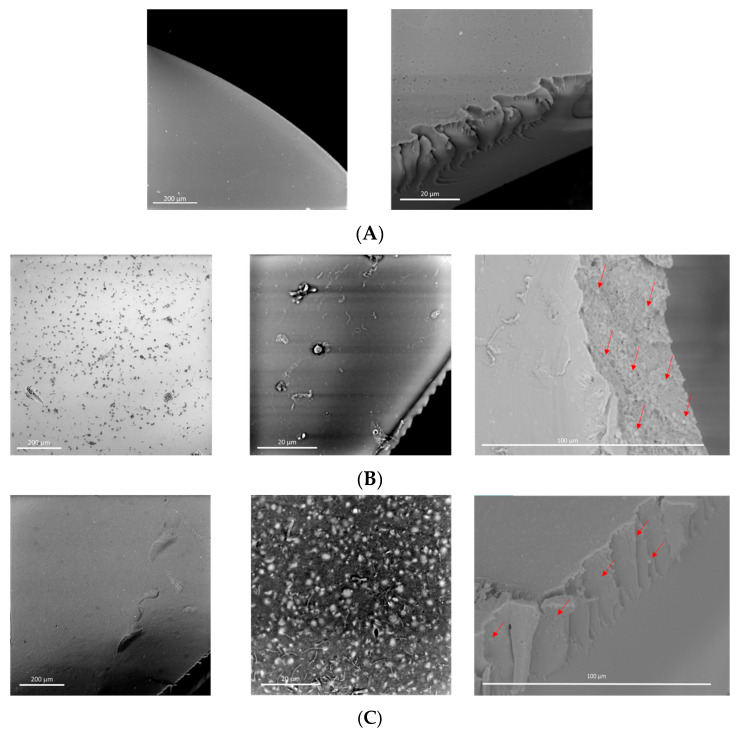
SEM micrographs of the membranes prepared from the polymer HMW-sPEES with (**A**) 0% MCF; (**B**) 6% MCF-NH_2_; and (**C**) 6% MCF-SO_3_H.

**Figure 13:**
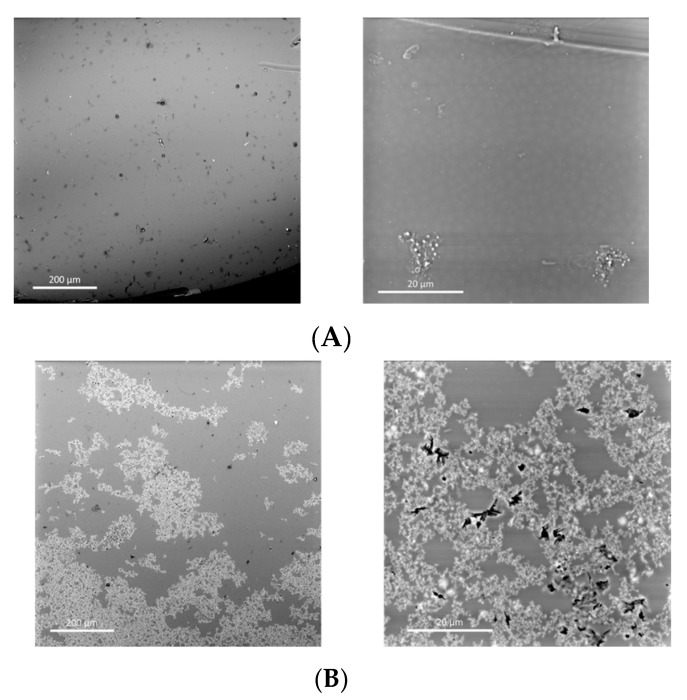
SEM micrographs of the membranes prepared from the polymer LMW-sPEES with (**A**) 6% MCF-NH_2_; and (**B**) 6% MCF-SO_3_H.

Finally, using scanning electron microscopy (SEM), the morphological properties of the HMW-sPEES membranes with 6% silica (Figure 12) and LMW-sPEES with 6% silica (Figure 13) were evaluated. The HMW-sPEES 0% Si membrane has a porous surface and a dense interior—or at least no pores are observed at the scale at which the image is presented. It is likely that if the system has pores inside the membrane they are below the scale at which the analysis was made.

In the case of HMW-sPEES 6% MCF-NH_2_ and HMW-sPEES 6% MCF-SO3H membranes (Figure 12B,C), particles (or aggregates of particles) are observed both on the surface and inside the membrane, showing that in the case of the HMW-sPEES 6% MCF-NH_2_ membrane, more particles are observed inside compared with the HMW-sPEES 6% MCF-SO3H membranes. However, in this last system, on the surface of the membrane, there is a better distribution of the particles. We observed a major formation of aggregates when comparing the images at the 200 µm scale.

For the membranes prepared from LMW-sPEES with 6% silica (Figure 13A,B), it is observed that for the LMW-sPEES 6% MCF-NH_2_ system, there are some regions with particles but they are not very dispersed throughout the membrane. In comparison, for the LMW-sPEES 6% MCF-SO_3_H membrane, there are many particles on the surface in the form of aggregates. In this case, microcracks are also observed on its surface, possibly caused by the aggregates of silica, which lead to the formation of stress points that fracture the membrane.

In all cases, these morphological differences will affect the functional properties of the membranes, thus affecting the capacity for water retention, proton exchange, and proton conductivity since a good dispersion of the inorganic material (silica) in the polymeric matrix (sPEES) is required. In this way, the interaction between both materials is favored, and therefore the final properties of the membrane are improved when compared with other membranes that do not have silica.

### 3.5. Water Uptake (WU)

The water retention capacity of a composite membrane (polymer–inorganic material) is one of the most important parameters because it affects the proton conductivity and mechanical stability of the membranes [76].

**Figure 14:**
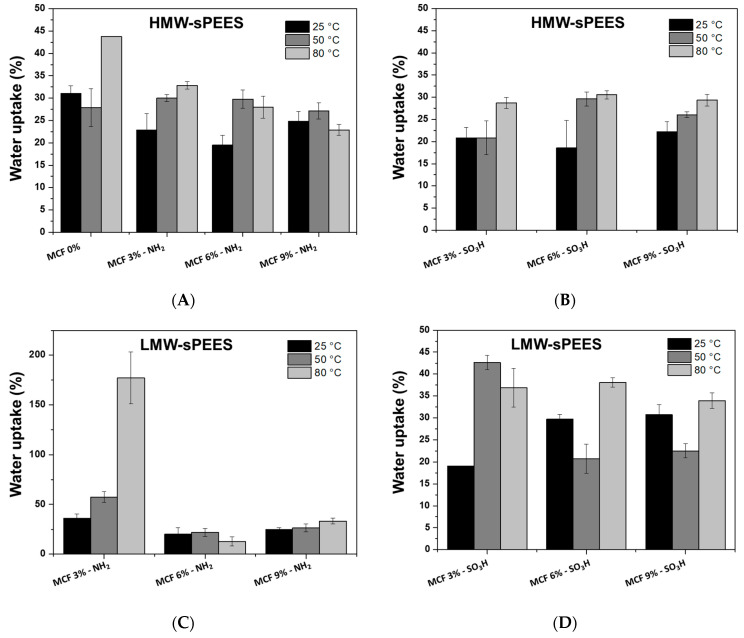
Measurements of water retention (%) at different temperatures for membranes prepared from (**A**) HMW-sPEES MCF-NH_2_; (**B**) HMW-sPEES MCF-SO_3_H; (**C**) LMW-sPEES MCF-NH_2_; and (**D**) LMW-sPEES MCF-SO_3_H.

Figure 14 shows the water retention values at different temperatures for the sPEES-silica composite membranes prepared from the HMW-sPEES and LMW-sPEES polymers. When comparing the results of the series of HMW-sPEES membranes with and without silica at different temperatures, it is observed that the membranes without silica (HMW-sPEES 0% MCF) present greater water retention at the three temperatures when compared with those that have silica (Figure 14A,B). This behavior has been reported for these polymer–inorganic material composite systems, in which the interaction between the polar groups of the polymer (-SO_3_H) and the silica particles (-NH_2_ or -SO_3_H) can reduce the sites of water absorption [77] and the mobility of the polymer chain, and thus the diffusion of water molecules through the membrane is reduced, thus decreasing water absorption [78].

In general, for these HMW-sPEES samples, it is observed that when the temperature increases from 25 °C to 80 °C, there is an increase in water retention; this behavior has been reported for other composite materials (polymer–silica) [31], which can be explained due to the thermal relaxation of the chains that favors the diffusion of water [73].

For the membranes prepared from LMW-sPEES, it was not possible to compare the systems with and without silica because the LMW-sPEES 0% Si membrane disintegrated easily, which prevented the measurement. However, when comparing the systems with different percentages of incorporated silica, it was again observed that when the temperature increases from 25 °C to 80 °C there is an increase in the water retention capacity. This increase is similar to the behavior observed for HMW-sPEES membranes with and without silica, which was previously explained.

### 3.6. Ionic Exchange Capacity (IEC)

**Figure 15:**
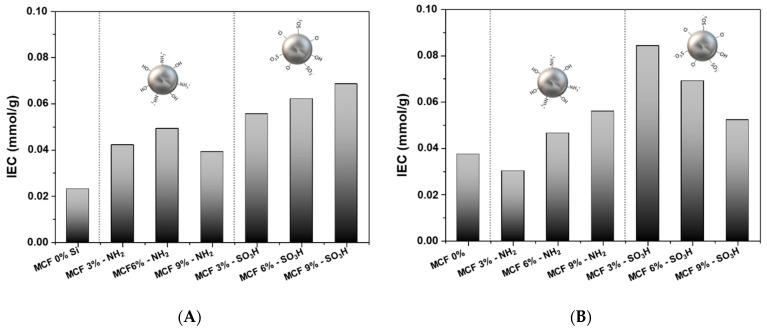
Measurements of ion exchange capacity (IEC) at 50 °C for membranes prepared from (**A**) HMW-sPEES and (**B**) LMW-sPEES.

The IEC measured in terms of the density of the ionizable hydrophilic groups present in the membrane provides a direct approximation of the conductivity of the proton [73]. Figure 15 shows the IEC for each of the prepared membranes. Both for the membranes prepared from HMW-sPEES and LMW-sPEES, an increase in the IEC is observed when incorporating silica (modified with amino groups and sulfonic groups) in the membrane. Additionally, as the silica content increases in the membrane, the IEC increases, except in the membranes prepared from LMW-sPEES with MCF-SO_3_H, where it was observed that the IEC decreases with the increase in silica content.

The increase in IEC with silica content may be related to a greater number of existing and available sites in the membrane to carry out ion exchange processes [77]; in this case, these sites are due to the presence of groups of the amino present in the silica (MCF-NH_2_) and to the sulfonic groups present both in the polymer and in the silica (MCF-SO_3_H).

Mainly, for the membranes prepared from LMW-sPEES, it is observed that the IEC for the three percentages of silica used is greater than that of the LMW-sPEES 0% Si membrane. However, the decrease in the IEC with the increase in the silica content may be due to an aggregation of the siliceous material as the silica content in the membrane increases, which instead of increasing the ion exchange sites decreases them, possibly due to preferential interactions between the same siliceous material which reduces the availability of sulfonic groups for ion exchange reactions. This aggregation could be observed for the LMW-sPEES 6% Si-SO_3_H sample through SEM analysis in Figure 13.

### 3.7. Proton Conductivity (σ)

**Figure 16:**
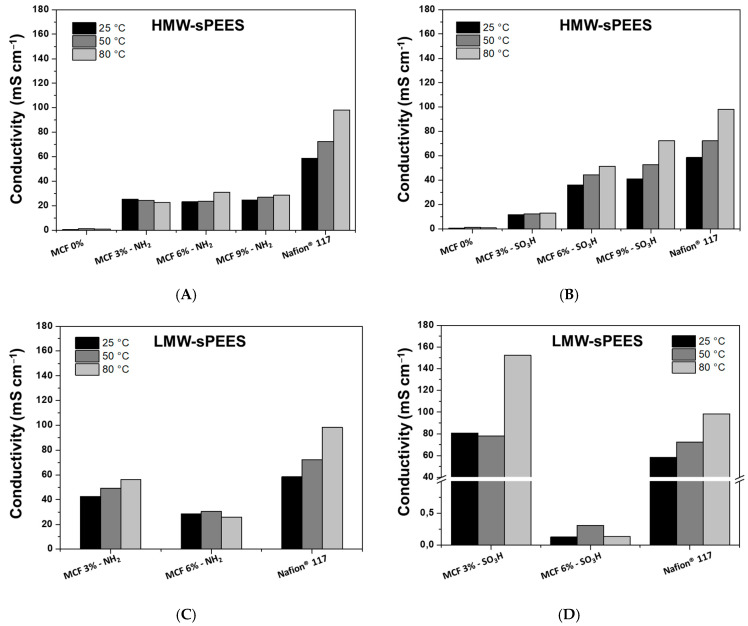
Proton conductivity measurements at different temperatures of membranes prepared from (**A**) HMW-sPEES MCF-NH_2_; (**B**) HMW-sPEES MCF-SO_3_H; (**C**) LMW-sPEES MCF-NH_2_; and (**D**) LMW-sPEES MCF-SO_3_H, and compared with Nafion^®^ 117.

Proton conductivity measurements of the membranes were carried out in the hydrated state and after being activated with H_2_SO_4_ for 3 days. The proton conductivity results as a function of temperature for the membranes prepared in this research and presented in Figure 16. The results were compared with the results of Nafion^®^ 117, which is considered to be the commercial reference material.

**Figure 17:**
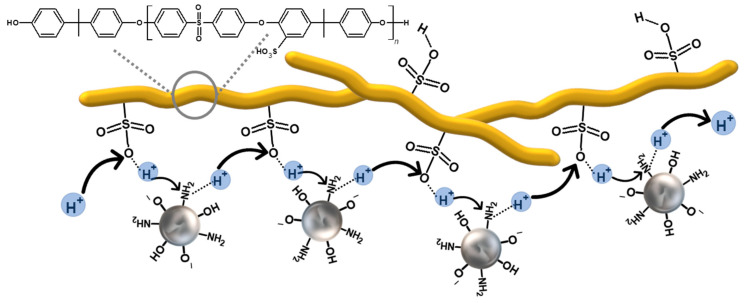
Proton transport in a Grotthuss mechanism proposed for sPEES membranes with modified silica with amino groups—through an acid–base interaction.

For the membranes prepared from HMW-sPEES with silica modified with amino groups (Figure 16A), it is observed that those systems that have silica have a higher proton conductivity compared with the membrane that does not have silica. This may be related to the formation of an acid-base pair between the amino groups of the silica and the sulfonic acid groups of the polymer [79]; this acid-base pair could serve as a ‘bridge’ that promotes proton transfer through the Grotthuss mechanism [45] (Figure 17). However, no substantial changes were observed for the different silica contents in the membranes, which is related to the IEC values obtained for these membranes (Figure 15A). Additionally, it is observed that the conductivities do not change as the analysis temperature increases, indicating that there is no dehydration or structural damage to the membranes as the temperature increases, which may suggest that the acid-base interaction is maintained in the system. However, these membranes have a lower proton conductivity compared with Nafion^®^ 117.

**Figure 18:**
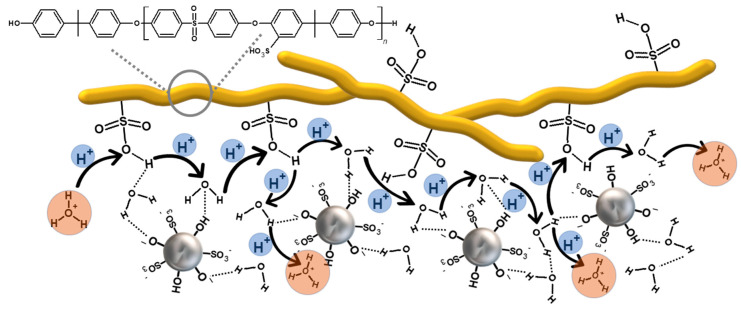
Proton transport in a proposed vehicular mechanism for silica-modified sPEES membranes with sulfonic groups—through hydrogen bonds.

Membranes prepared from HMW-sPEES with silica modified with sulfonic groups (Figure 16B) similarly show that there is an increase in proton conductivity by adding silica particles to the polymeric material. In this case, although not an acid–base pair, the formation of hydrogen bonds between water molecules with sulfonic groups is possible, potentially increasing proton transport through a vehicular mechanism [80] (Figure 18). When comparing the results of these materials with those of the same polymer but with silica modified with amino groups, it is observed that there is a higher proton conductivity for those whose silica has sulfonic groups, which correlates with the IEC values obtained for the latter membranes (Figure 18). There is likely higher mobility of the protons when the interaction is through hydrogen bonds rather than ionic interactions.

Additionally, it was observed that there is an increase in proton conductivity when increasing the analysis temperature of all these systems. This increase may be a consequence of the type of interaction that occurs between the silica and the polymer, where an increase in temperature favors the mobility of polymer chains, and thus proton transport across membranes is increased. Finally, the HMW-sPEES 9% Si-SO_3_H membranes have the highest conductivity of all the systems based on HMW-sPEES and would be the most comparable system with the conductivity of Nafion^®^ 117.

The membranes prepared from the LMW-sPEES polymer (lower molecular weight) presented lower mechanical stability (qualitative), and the samples with 9% silica (modified with amino groups and sulfonic groups) fractured before making the proton conductivity measurement. Therefore, these values are not shown in Figure 16C,D. For these systems, it was observed that the membranes with 3% silica have a higher conductivity when compared with the 6% samples—both for those modified with amino groups and with sulfonic groups. This result could be related to the formation of aggregates of silica particles that are formed by increasing the content in the membranes, as observed in the SEM images (Figure 13). However, the samples with 3% silica showed acceptable proton conductivity values for this application. The LMW-sPEES 3% Si-SO_3_H membrane was the one that presented a higher proton conductivity compared with all the systems studied in this investigation, including Nafion^®^ 117. This result allows concluding that this material is a possible alternative to be used as a proton exchange membrane in fuel cells.

## 4. Conclusions

PEES polymeric matrixes of different molecular weights (LMW-PEES: 7600 Da and HMW-PEES: 15,200 Da) were obtained and modified by incorporating sulfonic groups in the aromatic rings of the polymer. The high surface area, large pore size, and volume MCF-type mesoporous silica particles were obtained through a sol-gel method. The modification of the silica particles with amino groups and sulfonic groups was achieved. The membranes prepared by the solvent evaporation method were characterized based on their ability to retain water, ion exchange capacity, and proton conductivity.

We found that membranes made with both polymers incorporating 3% and 6% of silica preserved their physical integrity. However, generally, the membranes prepared from the polymer with high molecular weight were more suitable to manipulation. Concerning the effect of functionalization in the silica particles, the type of functional group incorporated impacted the ion exchange capacity and, therefore, the proton conductivity was related to the different kinds of interaction that occur between the modified silica and the sulfonic groups in the polymer. In the case of silica with amino groups, an acid–base pair is formed, promoting proton transport along the membrane through a Grotthuss mechanism. In contrast, in silica with sulfonic groups, the proton transport through the membrane can be influenced by the interaction through hydrogen bonds that occurs between the polymer and the silica, which can favor a vehicular mechanism for proton transport.

Finally, it was found that the membranes with silica had better functional properties than those without; additionally, the percentage of incorporated silica affected the ion exchange capacity and proton conductivity. Specifically, membranes based on the modified silica with sulfonic groups incorporated into the low molecular weight polymer (LMW-sPEES) at 3% had more excellent conductivity when compared with Nafion^®^ 117, which is the commercial reference material. Table 5 presents a comparison of the membrane with the best performance in terms of conductivity prepared in this work, in relation to the Nafion membrane, which is the reference membrane. For the conductivity property of the Nafion, in this work, the measurements of this sample were also carried out since the conductivity depends on whether it is evaluated in the plane or through the plane. The results as a whole show a good performance of the prepared membranes, for which it can be concluded that this system becomes a potential membrane as a proton exchange membrane in fuel cells.

## Figures and Tables

**Table 1:** Summary of the properties of some composite membranes reported in previous works.

Sample	Proton Conductivity	Ionic Exchange Capacity	Methanol Permeability	Water Retention	Reference
Nafion 117	0.113 S/cm at 25 °C	0.93 meq/g	8.84 × 10^−7^ cm^2^/s (60 °C 5M)	30% at 30 °C	[39]
F-GO/Nafion membrane: functionalized GO nanosheets (F-GO) with a sulfonic acid functional group (3-mercaptopropyl trimethoxysilane)	0.012–0.047 S/cm at 120 °C	0.96 meq/g (5% F-GO) and 0.93 meq/g (10% F-GO),		~25% (5% F-GO) and ~29% (10% F-GO)	[8]
Sulfonated poly(arylene ether sulfone) (SPAES) composite membranes with graphene oxide (GO) and sulfonated poly(arylene thioether sulfone)-grafted graphene oxide (SATS-GO) as fillers	131.43 mS/cm SPAES/ SATS-GO-2.0 at 80 °C and 90% RH			77.7% at 80 °C	[28]
Nafion–TiO_2_ (9%)	0.1–0.15 (*10^−2^ S/cm) (50–130 °C)		7.91% (2 M) and Nafion 9.9%	17.77%	[33,40]
Nafion CS-SiO_2_ 6%	0.17 S/cm at 80 °C	Around 0.96 meq/g	Around 6 (*10^−7^ cm^2^/s) (1 M)	30%	[33,41]
Sulfonated SBA-15 mesoporous silica (SM-SiO_2_)-incorporated sulfonated poly(- phenylsulfone) (SPPSU) composite membranes	5.9 mS/m 80 °C and 50% RH			252% at 80 °C	[36]
Sulfonated poly(ether ether ketone) (SPEEK) doped with silica sulfuric acid (SSA)	0.13 S/cm at 80 °C (5 wt.% SSA) (Nafion 0.12 S/cm)	1.25 mmol/g		0.56% at 80 °C	[29]
Cross-linked sulfonated poly(arylene ether ketone) with silica nanoparticles (CL-SPAEK/silica)	3.06 mS/cm, (SPAEK: 0.32 mS/cm) at 70 °C under 30% RH	1.75 meq/g		Around 56% at 90 °C	[31]

**Table 2:** Assignment of the positions and number of protons present in the structure of the sulfonated polymers.

**Position**	2 + 2′	2″	3 + 3′
**Proton number**	4-*DS*	*DS*	4

**Table 3:** Surface area, mesoporous volume, pore size, and particle size of MCF silica.

Sample Name	Surface Area ^1^ (m^2^/g)	Mesoporous Volume (cm^3^/g)	Pore Size ^2^ (nm)		Particle Size ^3^ (nm)
			D_c_	D_w_	
MCF silica	797.6	2.17	21.95	10.90	610.5

^1^ Surface area: specific surface area determined by adsorption–desorption of nitrogen. ^2^ Pore size obtained from the maximum of the peak of the pore size distribution curve for adsorption (D_c_) and desorption (D_w_). ^3^ Particle size determined by DLS.

**Table 4:** Surface area, pore volume, and pore size for MCF silica before and after functionalization with APTES (MCF-NH_2_) and MPTMS (MCF-SO_3_H).

Sample	BET Area (m^2^/g)	Pore Volume (cm^3^/g)	Cell Size (nm)	Window Size (nm)
**MCF**	774.98	2.08	20.39	10.63
**MCF-NH_2_**	560.65	1.70	20.64	9.86
**MCF-SO_3_H**	649.47	1.64	19.77	9.89

**Table 5:** Summary of the performance properties of the LMW-sPEES with MCF 3%-SO_3_H membrane and Nafion 117.

Sample	Proton Conductivity	Ionic Exchange Capacity	Water Retention	Reference
Nafion^®^ 117	113 mS/cm at 25 °C	0.93 meq/g	30% at 30 °C	[39]
LMW-sPEES + MCF 3%-SO_3_H	80 mS/cm at 25 °C 80 mS/cm at 50 °C 160 mS/cm at 80 °C	0.08 mmol/g	20% at 25 °C 42% at 50 °C 36% at 80 °C	This work
Nafion^®^ 117	60 mS/cm at 25 °C 70 mS/cm at 50 °C 100 mS/cm at 80 °C	---	---	This work

## Data Availability

The data presented in this study are available on request from the corresponding author.
